# Research progress of nanovaccine in anti-tumor immunotherapy

**DOI:** 10.3389/fonc.2023.1211262

**Published:** 2023-08-24

**Authors:** Min Yao, Xiyu Liu, Zhangbo Qian, Dianfa Fan, Xinjun Sun, Liping Zhong, Pan Wu

**Affiliations:** ^1^ State Key Laboratory of Targeting Oncology, National Center for International Research of Bio-targeting Theranostics, Guangxi Key Laboratory of Bio-targeting Theranostics, Collaborative Innovation Center for Targeting Tumor Diagnosis and Therapy, Guangxi Medical University, Nanning, Guangxi, China; ^2^ Pharmaceutical College, Guangxi Medical University, Nanning, Guangxi, China

**Keywords:** anti-tumor cells, nanovaccine, nanocarrier, tumor antigen, adjuvant

## Abstract

Tumor vaccines aim to activate dormant or unresponsive tumor-specific T lymphocytes by using tumor-specific or tumor-associated antigens, thus enhancing the body’s natural defense against cancer. However, the effectiveness of tumor vaccines is limited by the presence of tumor heterogeneity, low immunogenicity, and immune evasion mechanisms. Fortunately, multifunctional nanoparticles offer a unique chance to address these issues. With the advantages of their small size, high stability, efficient drug delivery, and controlled surface chemistry, nanomaterials can precisely target tumor sites, improve the delivery of tumor antigens and immune adjuvants, reshape the immunosuppressive tumor microenvironment, and enhance the body’s anti-tumor immune response, resulting in improved efficacy and reduced side effects. Nanovaccine, a type of vaccine that uses nanotechnology to deliver antigens and adjuvants to immune cells, has emerged as a promising strategy for cancer immunotherapy due to its ability to stimulate immune responses and induce tumor-specific immunity. In this review, we discussed the compositions and types of nanovaccine, and the mechanisms behind their anti-tumor effects based on the latest research. We hope that this will provide a more scientific basis for designing tumor vaccines and enhancing the effectiveness of tumor immunotherapy.

## 1 Introduction

Cancer vaccines usually combine exogenous tumor antigens with adjuvants or dendritic cells (DCs) themselves to activate DCs, thereby stimulating strong anti-tumor humoral or cellular immunity. Therapeutic tumor vaccine harnesses the patient’s immune system to activate antigen-specific cascade immune responses and ultimately achieve the killing effect on tumor cells, establish durable immune memory, and reduce nonspecific or adverse effects (1). The basic principles of successful therapeutic cancer vaccine administration include delivering a large number of high-quality antigens to DCs, optimal activation of DCs, induction of robust and sustained CD4^+^ T cell and cytotoxic T lymphocyte(CTL) responses, infiltration of the tumor microenvironment (TME), and persistence and maintenance of the immune response. The use of tumor vaccines for treating established malignancies has a long history dating back to the 1910s when William B. Coley, an American orthopedic surgeon, injected sarcoma patients with killed streptococci and Serratia marcescens (2). He observed significant tumor suppression and immune enhancement effects resulting from the treatment. In the late 20th century, Ellis L. Reinherz conducted a lot of research on T cell receptors (TCRs) and how TCRs bind to peptide-loaded major histocompatibility complex (MHC), which has inspired the rational design of tumor vaccines (3–5). Sipuleucel-T was the first Dendritic cell-based vaccine to receive approval from the Food and Drug Administration (FDA), demonstrating significant overall survival benefits for prostate cancer patients (6). However, the heterogeneity and low immunogenicity of tumors, as well as the presence of immune escape mechanisms, inhibit the effectiveness of tumor vaccines (7). Fortunately, the rapid development of biotechnology and materials science has led to the emergence of tumor nanovaccines. Tumor nanovaccines utilize nanoparticles (NPs) as carriers to deliver tumor antigens and immunomodulators, thereby activating the immune system to generate a targeted immune response against the specific tumor. Tumor nanovaccines possess numerous advantages compared to conventional tumor vaccines. (a) Encapsulation of antigens in nanocarriers can prevent antigens from degradation, provide a stable delivery platform, prolong the continuous release time of antigens *in vivo*, and enhance the durability of immune responses (8). (b) Nanovaccines can carry diverse antigens and adjuvants at the same time to achieve multiple immune stimulations to enhance immunogenicity and therapeutic immune responses (9). (c) The size (10) and shape (11) of NPs can be precisely controlled to adapt to different cargoes, thereby improving vaccine stability and immune activity. (d) Surface modifications of NPs by targeting ligands or changing the surface properties enable targeted delivery to lymphoid tissues (12) and antigen-presenting cells (13)(APCs), improving the efficiency and selectivity of vaccines. (e) Some NPs are designed for cytoplasmic delivery of antigens to facilitate the process of antigen cross-presentation and thereby enhance the anti-tumor response of CTLs (14). (f) Some nanocarriers have the properties of immune adjuvant, which can deliver tumor antigens and stimulate the body’s immune system at the same time (15). More details are summarized in **Table 1**.

**Table 1:** Comparison of conventional and nanovaccines.

Conventional vaccines	Disadvantage	Nanovaccines	Improvement	References
Attenuated live vaccine or inactivated vaccine	Risk of causing disease	Subunit vaccines (i.e., protein, peptide, mRNA, or DNA)	Safe and economical	(16–20)
Mix antigen and adjuvant in the medium	Low bioavailability and adverse injection events	Encapsulate antigen and adjuvant with nanocarriers	Protect the bioactivity of antigen and adjuvant	(21, 22)
Antigen and adjuvant are separate parts	Poor colocalize	Co-delivery and colocalize antigen and adjuvant as a unit	Improve immunogenicity	(23, 24)
	Lack of targeting	Small size, adjust physical and chemical properties and modify surface with functional ligands	Enable efficient lymph node drainage and target specific cell subsets or TME	(25–33)

Hence, in this review, we present the research progress of nanovaccines and discuss the opportunities and challenges of nanovaccines in tumor immunotherapy. We will discuss the composition of nanovaccines and the mechanism of nanovaccines in enhancing T-cell responses to cancer immunotherapy.

## 2 Composition of nanovaccines

Nanovaccines consist of three main components: (1) antigens, (2) adjuvants, and (3) nanocarriers. The antigens can be derived from natural tumor antigens (34), synthetic peptides (35), mRNA (36), and DNA (37) encoding tumor antigens, which are the source of tumor immunogenicity (36, 38). Studies have shown that when used alone, subunit vaccines have difficulty producing sufficiently strong and durable protective immunity against pathogens due to their weak immunogenicity (39). To enhance the immunogenicity of high-purity vaccine antigens, the addition of adjuvants without compromising vaccine safety to the formulation has been a major goal in vaccine design (40). As for conventional vaccines, antigens and adjuvants are separate components, which may lead to adjuvants wastage, reduced vaccine immunogenicity, and an increased risk of adverse immune reactions. Nanoparticle-based delivery vehicles can facilitate the co-delivery of antigens and adjuvants as a whole, which significantly improves the efficacy and safety of vaccines and achieve targeted delivery (41). For tumor vaccines, nanomaterials serve as a bridge to efficiently deliver antigens and adjuvants to the target sites, thereby stimulating effective immune responses.

### 2.1 Tumor antigens

Tumor antigens are produced by genetic mutations or abnormal or faulty protein translation modifications (42). They are either tumor-specific expressed, which means they are not present in normal tissue cells and are called tumor-specific antigens(TSAs) (43), or present in the normal body but are overexpressed in cancer cells, called tumor-associated antigens(TAAs) (44). According to the different sources of tumor antigens, nanovaccines are mainly categorized into four types: peptide vaccines, DNA vaccines, mRNA vaccines, and cell vaccines.

The abundance of mutations observed in tumor genomes can be leveraged as a valuable resource for the development of tumor vaccines. Neoantigens are mutant peptides resulting from gene mutation transcription and translation that can specifically induce T lymphocytes (45). Neoantigens are highly specific and immunogenic, have no thymus-negative screening, and are expressed only in tumor tissues (43). Therefore, tumor neoantigens are considered ideal targets for immunotherapy, especially for individualized tumor vaccines. To identify neoantigens, whole genome or exonic sequencing of somatic and tumor cells from the same patient is required to recognize mutations in individual tumor protein-coding genes (46). Not all mutant peptides are neoantigens; only those that can be recognized and presented by MHC and elicit a T-cell immune response can be called neoantigens. Many clinical trials of personalized therapeutic cancer vaccines based on neoantigens are ongoing. In an ongoing phase II trial in patients with advanced non-small cell lung cancer, four aqueous peptide mixtures were synthesized, each containing six neoantigen peptides. Extensive stability studies at −25°C or −80°C showed that the formulation was stable for up to 32 weeks, which provided guidance for the development of new personalized therapeutic vaccines (47). However, screening neoantigens is a complex process, different databases or algorithms may produce different results, and it is necessary to comprehensively consider the time, tool, labor costs, and treatment benefits.

#### 2.1.1 Peptide antigens

Peptide vaccines contain the key amino acid sequences of tumor antigens, which can precisely activate the immune response, are easy to produce, and have good safety. To be effective, peptide vaccines must have CD8^+^ T cell epitopes to activate cytotoxic CTL anti-tumor immunity through the antigen cross-presentation pathway and CD4^+^ T cell epitopes to activate T helper cells (48). Multi-epitope vaccines have emerged as a promising strategy for immunotherapy of cancer. In a study, researchers used the early proteins E5, E6, and E7 of human papillomavirus type 16 as target antigens to form a novel multi-epitope vaccine (E765m) with peptides E5aa28-46, E6aa37-57, and E7aa26-57, which was inserted into the main immunodominant region of the hepatitis B virus core antigen to construct chimeric virus-like particles(VLPs) HBc-E765m. The results showed that the peptide vaccine could induce strong cellular and humoral immunity (17).

#### 2.1.2 DNA encoding tumor antigens

DNA vaccines are plasmids designed to deliver genes encoding tumor antigens, inducing or enhancing adaptive immune responses against tumor cells carrying the tumor antigens (49).DNA vaccines have the potential to provide a broad range of tumor antigens and are relatively easy to prepare and preserve compared to traditional tumor vaccines (38). Since the antigens are synthesized and expressed by the body’s cells, they can be continuously presented to the immune system, potentially leading to long-term immune responses. In phase I/IIa trial of a therapeutic DNA vaccine against HPV16-positive high-grade cervical intrae-pithelial neoplasia(CIN), E6 and E7 antigens were delivered directly to APCs by targeting CCR5, the receptor for the chemokine CCL3L1, to increase antigen loading and cross-presentation. The results showed that in the expansion cohort, HPV16 clearance was seen in about half of the evaluable subjects, the majority of the lesions shrank, and more than half of the lesions regressed to CIN 0/1 (50). DNA vaccines are easy to produce quickly (51), do not require dealing with dangerous pathogens (52), are thermostable, and facilitate low-cost storage and transportation (53). However, a major limitation of candidate DNA vaccines delivered through needles and syringes is poor immunogenicity, resulting in low efficiency of cell uptake of DNA (54). Therefore, the development of safer and more effective delivery systems is needed.

#### 2.1.3 mRNA encoding tumor antigens

mRNA encoding specific tumor antigens or full-length tumor antigens can also be used as a source of tumor antigens to initiate tumor recognition by the immune system with the advantages of high efficiency and safety, as well as economic cost (55). mRNA delivery does not require crossing the nuclear membrane because it is translated into protein by ribosomes in the cytoplasm (56). This means that there is no risk of genomic integration or insertional mutations, which can be a concern with other gene therapy approaches that require nuclear delivery (57). Nevertheless, the application of mRNA vaccine technology is significantly limited by challenges including low *in vivo* stability and delivery efficiency (58). Appropriate mRNA structural modifications (59), tweaked delivery routes (60), and codelivery of vaccines containing other immunotherapeutic agents (61)are expected to increase the likelihood of tumor cell eradication.

#### 2.1.4 Tumor cells as tumor antigens

Tumor cell lysates (TCLs) can provide a bunch of repertoire of TAAs without the need to identify and synthesize optimal antigens for specific types of cancer. DCs-based nanovaccine loaded with different TAAs plays a key role in immunotherapy and immunoprophylaxis. Lu’s group reported a hydrogel vaccine system containing tumor lysates and granulocyte-macrophage colony-stimulating factor (GM-CSF). The GM-CSF was released from the hydrogel to recruit DCs to provide a fully personalized tumor antigen pool. The results showed that the personalized hydrogel implanted into the surgical site could stimulate the anti-tumor immune response of the body and inhibit residual tumor cells (62). Fusing tumor cells with DCs is also a promising immunotherapy strategy. These fusion cells can simultaneously express tumor-specific antigens and present co-stimulatory signals, activating the body’s immune system (63). Although whole tumor antigens have demonstrated their utility in eliciting sustained CTL responses and vaccine effectiveness in cancer therapy, they often fail to induce sufficiently strong CTLs anticancer responses and require combination with other immune agonists or adjuvants. In one study, the immunologically dying tumor cells and DCs were co-delivered to mice with the immune adjuvant cytosine-phosphate-guanine (CpG) by a hydrogel delivery system. The results showed that relative to living tumor cells, immunologically dead tumor cells effectively activated DC maturation with the aid of the immune adjuvant CpG nanoparticles (64).

### 2.2 Nanocarriers

Nanocarriers are promising tools to carry multiple therapeutic agents, increase the solubility of cargo, stabilize therapeutic activity *in vivo*, enhance the targeted delivery of drugs, and reduce drug-related side effects (65). Some carriers possess adjuvant properties, which activate the immune system and enhance immune responses, thereby improving the efficacy of vaccines (66). Liposomes, polymer nanocarriers, inorganic nanocarriers, and biomimetic nanocarriers are among the commonly used nanocarriers today.

#### 2.2.1 Liposomes

Liposomes are spherical vesicles composed of lipid bilayers, primarily made from natural lipid molecules such as lecithin (67) and cholesterol (68). Their particle size typically ranges between 1nm and 10 μm. Liposomes exhibit a structure similar to that of cell membranes, making them highly biocompatible. They offer the ability to encapsulate within their internal aqueous phase (69), while lipophilic drugs can be dispersed within the lipid bilayer (70). This improves drug stability, enhances bioavailability, and reduces drug toxicity. Moreover, the surface of liposomes can be modified with proteins or peptides to achieve specific targeting by binding to target cells (31). However, liposomes possess certain limitations that need to be addressed. Some anti-tumor drugs have low encapsulation efficiency within liposomes, and these drugs may easily leak out, thereby reducing the efficacy of modification or encapsulation (71). Furthermore, the stability of liposomes under improper manufacturing or unsuitable storage conditions shows great challenges to the commercialization process. In one study, about 40% of the contents were shown to leak from conventional liposomes when stored in phosphate buffer (PBS) (72).

#### 2.2.2 Polymer nanocarriers

Polymeric microparticles such as poly lactic-co-glycolic acid (PLGA) are extensively employed as drug delivery systems for cancer immunotherapy. PLGA enables the encapsulation of both hydrophilic and hydrophobic drugs, as well as organic biological molecules like peptides (73), nucleic acids (74), and polysaccharides (75). It was reported that PLGA NPs carrying tumor antigens and adjuvants can induce cellular immunity and recruit T cells to the tumor site for effective anticancer immunotherapy (76). PLGA exhibits excellent biodegradability, where its ester bonds eventually break down into metabolizable monomers such as lactic acid and glycolic acid within the body (77). Moreover, the size, stability, and solubility of PLGA NPs can be effectively adjusted (78). However, the complexity of the manufacturing process, the precise release of the drug, and its *in vivo* stability all need to be considered.

#### 2.2.3 Inorganic nanocarriers

Inorganic NPs offer multipurpose platforms for various delivery applications. Inorganic nanoparticle-based nanomedicine has witnessed significant advancements over the past few decades, owing to its multifunctionality, tuneability, and unique physical and chemical properties (79). Liu et al. developed a gold nanorod with excellent photothermal efficiency and stability. It can effectively absorb tumor-derived protein antigens and directly form a nanovaccine *in vivo (* 80). This nanovaccine enhances the activation of host DCs, thereby amplifying the adaptive antit-umor T cell response, triggering an effector memory immune response, and activating innate anti-tumor immunity. Mesoporous silica NPs are an attractive delivery platform due to their mesopores, high specific surface area, large pore volume, easy-to-modify physical and chemical functions, biocompatibility, and self-adjuvantability (81, 82). Gao et al. prepared virus-like mesoporous silica NPs by mimicking the surface structure, centripetal-radialized spike structure, and rough surface topology of viruses, which induced a robust cellular immune response with higher cell invasion ability and a unique endocytic pathway, and could induce effective tumor immune responses and inhibit tumor growth (83).

#### 2.2.4 Biomimetic nanocarriers

Biogenic NPs refer to naturally produced NPs by living organisms or derived from their cellular components and have gained increasing interest as carriers for displaying cancer antigens in the field of tumor vaccines. These nanoparticles can be generated through various biological processes, such as the release of outer membrane vesicles (OMVs) by bacteria, the secretion of exosomes by eukaryotic cells, or the expression of viral structural proteins to form virus-like particles. OMVs contain a large number of immunogenic components related to their parent bacteria, which can be used as vaccines, adjuvants, and vectors to treat diseases, especially in tumor antigen presentation or small molecule drug targeted therapy (84). Exosomes are lipid bilayer extracellular vesicles secreted by all types of cells, carrying large amounts of nucleic acids, proteins, and other bioactive substances which can be transferred into recipient cells as functional components in exosomes (85). Exosomes, as nanocarriers, possess attractive pharmacokinetic and immunological characteristics. They offer the advantage of penetrating physiological barriers that synthetic drug carriers are unable to traverse. Unfortunately, the isolation and purification of exosomes still pose significant technical challenges. VLPs are protein complexes that self-assemble to resemble the structure of native viruses, but they cannot cause infection (86). VLPs possess notable advantages as vaccine carriers owing to their well-defined chemical composition, structured spatial arrangement, excellent biocompatibility, and considerable potential for clinical application (87). VLPs are generally multivalent structures, which can stimulate strong humoral and cellular immunity (17). Overall, despite the many advantages of VLPs as vaccine vectors, their application still faces several technical, manufacturing, and safety challenges.

### 2.3 Immunologic adjuvants

Immune adjuvants, also known as immune boosters, are key components of vaccines that significantly enhance the magnitude, breadth, and persistence of the immune response (88). The immunostimulatory properties of adjuvants are essential for enhancing the immunogenicity of subunit antigens (89). Adjuvants can be classified into two categories according to their mechanism of action, vaccine delivery systems and immunostimulants. Mineral salts, emulsion adjuvants, liposomes, and biogenic nanomaterials, and are commonly used as vaccine delivery systems to more effectively present antigens to effector cells, achieve controlled antigen release, and induce long-lasting immunity. Immune adjuvants can activate innate immunity either by acting directly as cytokines or indirectly through the recognition of specific patterns by pattern recognition receptors(PRRs) (90, 91). The main types and functions of immune adjuvants are summarized in **Table 2**. Although adjuvants are extremely critical for the efficacy of most vaccines, they also have the potential to induce undesirable immune reactions. Therefore, how to achieve an efficient combination of antigenic adjuvants while producing minimal side effects is a factor to be considered before vaccine design.

**Table 2:** The main types and functions of immune adjuvants.

Type	Adjuvant	Function	References
Delivery systems	Calcium phosphate	Biodegradable, low toxicity, and high loading capacity	(92)
	Aluminum salts	Enhance humoral immunity, and prolonged the intratumoral retention time of drugs	(93)
	Emulsions	Form a depot at the injection site, induces inflammation, and gradually release the antigen	(94)
	Liposomes	Similar to the structure of biofilm, deliver drugs or antigens into targeted cells, and have high bioavailability	(95–97)
	Metal	Enhance immunogenicity and cellular uptake	(80, 98)
	Non-metallic material	High cargo-carrying capacity and unique physicochemical properties can be combined with physical therapy	(99, 100)
	Biogenic nanomaterials	Induction of sufficient anti-tumor responses without causing significant adverse effects	(17, 101–103)
Immunostimulants	TLRa	Detecting PAMPs and responding to them by activating innate and adaptive immune pathways	(83, 95, 98, 104–107)
	Cytokines	Promote DCs maturation and subsequently enhance the generation of antigen-specific CD8^+^ T cell	(93, 108)
	Chemokines	Regulate lymphocyte development, initiate and execute effector functions, and enhance the capacity for protective immunity	(109, 110)
	STING agonists	Ensure the production of type I interferon to assist in regulating the immune activity	(111)
	Carbohydrate-based adjuvants	Transfer information and stimulate humoral and cellular immunity	(94, 95, 98, 112)

## 3 Mechanism of action of nanovaccine

**Figure 1:**
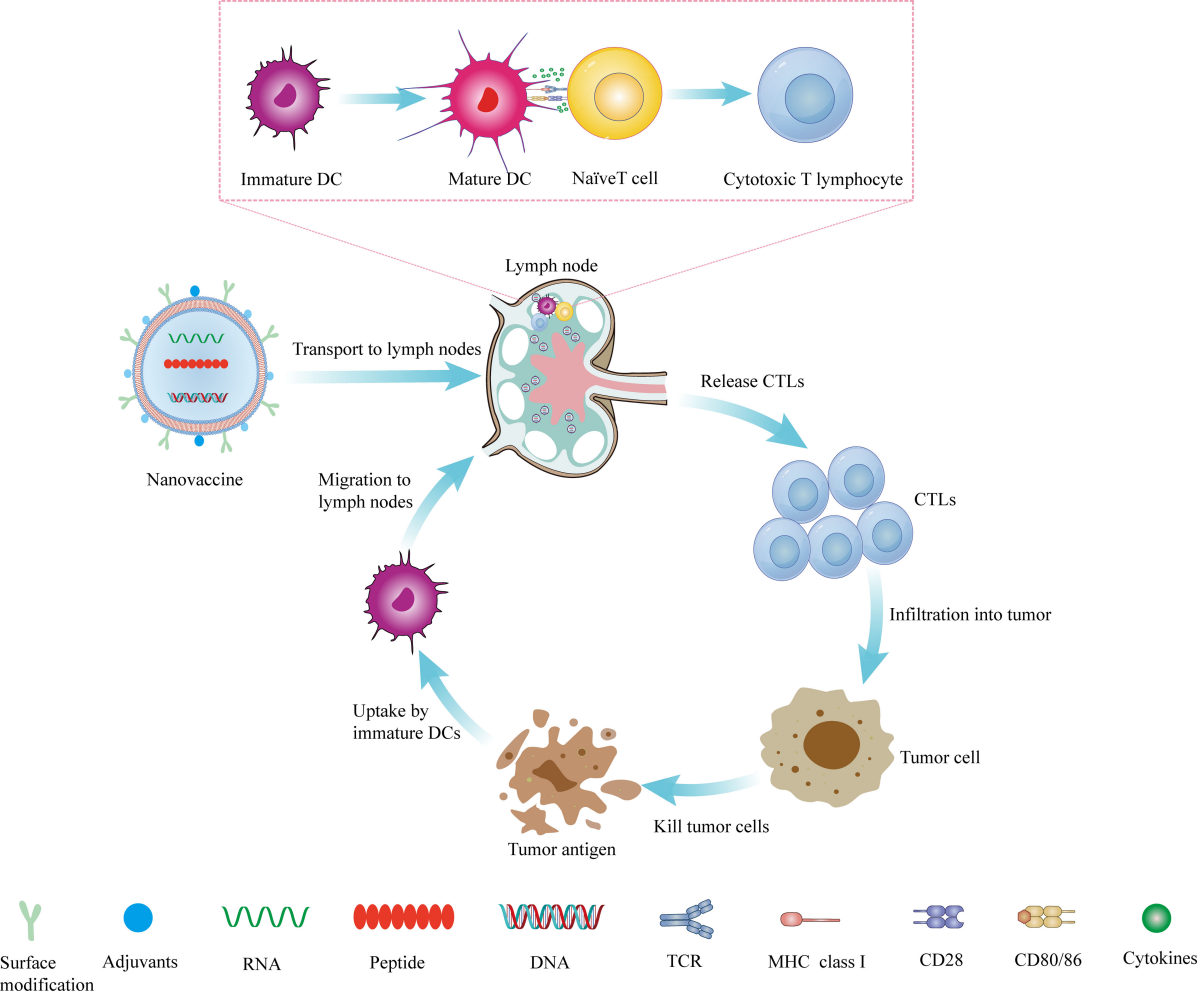
The mechanism of nanovaccine. The nanovaccine drains to the lymph nodes, where it stimulates the production of CTLs, and then infiltrates the tumor site from the efferent lymphatic vessels. It kills tumor cells and produces tumor antigens, which can be captured and presented by DCs. DCs migrate to lymph nodes to stimulate the production of T lymphocytes and reactivate the body’s anti-tumor immunity.

Although nanovaccines have many advantages over conventional vaccines, there are still challenges that stand in the path of their clinical application. Nanovaccines need to undergo a five-step cascade reaction to exert their anti-tumor effects *in vivo*: loading of tumor-specific antigens by the nano-delivery system (L), priming of tumor antigens to lymph nodes (D), internalization by DCs (I), stimulation of DCs maturation (M), and presentation peptide-MHC I complexes to CD8^+^ T lymphocytes (P) (referred to as the LDIMP cascade) (113).To achieve an effective cell-mediated immune response, nanovaccines need to be carefully designed and optimized at each step to achieve a synergistic immunotherapy effect (**Figure 1**).

### 3.1 Improve immunogenicity

The effectiveness of tumor vaccines is strongly associated with their immunogenicity (114). However, endogenous antigens produced by immunogenic death are often inadequate to elicit robust anti-tumor immunity. The low immunogenicity of tumors is one of the major bottlenecks of tumor immunotherapy. Enhancing tumor immunogenicity can improve the efficacy of tumor immunotherapy by increasing tumor antigen exposure and DCs targeting, and supplementing with adjuvant (115).

The delivery of exogenous immunogenic antigens and the release of endogenous immunogenic antigens by inducing immunogenic cell death (ICD) are the two main ways to increase antigen exposure. In the former case, these immunogenic antigens can be peptides (116), TCLs (32), nucleic acids (117), and neoantigens (112). In the latter case, inducing ICDs in tumor cells, resulting in the release of various danger-associated molecular patterns (DAMPs) and TAAs, can effectively stimulate anti-tumor immune responses, promote DC maturation, and facilitate the infiltration of CTLs (104).

Receptors exist on the surface of DCs that mediate the uptake of extracellular antigens and their import into the phagosomal compartment of DCs. Therefore, direct targeting of antigens via nanocarriers to specific extracellular receptors expressed by DCs, such as DEC-205, CLEC9a, XCR1, and mannose receptors (118), is an effective strategy to enhance immunogenicity. Feng et al. utilized nanoliposomes to encapsulate OVA and dulcimer, which were targeted to DCs using anti-DEC-205 receptor antibodies (30). The study found that the nanoliposomes enhanced the proliferation and maturation of DCs, improved their phagocytic efficiency, and stimulated both cellular and humoral immunity. In some cases, adjuvants such as cytokines, chemokines, TLR agonists, and STING agonists are co-administered with tumor antigens to further enhance the immunogenicity of tumors. CLEC9a is a C-type-lectin-like receptor that is specifically expressed in DCs. CLEC9a antibodies induce humoral, CD4^+,^ and CD8^+^ Tcell responses through antigen-specific delivery to DCs. Researchers constructed a 12-peptide vector CBP-12 with high affinity to Clec9a to deliver exogenous or endogenous antigens (119). Following this work, a nanovaccine drug delivery system coated with an engineered peptide expressing bionic cancer cell membrane (EPBM) was designed (PLGA/STING@EPBM) to deliver STING agonists and tumor antigens to Clec9a(+)DC, which significantly enhanced IFN-stimulated gene expression and antigen-cross-presentation of Clec9a(+)DC, resulting in a strong anti-tumor effect in both anti-PD-1 responsive and drug-resistant tumor models (120). Wylie and colleagues constructed a novel vaccine containing XCL1 ligands to target the uptake of XCR1(+) cross-presenting DCs and a cell-penetrating peptide (CPP) with endosome escape properties to enhance antigen entry into the cross-presenting pathway, which showed promising tumor suppression in a B16 murine melanoma model (13). Similarly, DC-targeting mannose and immune adjuvant CpG-ODN were assembled on the surface of liposomes, loaded with melanoma-specific TRP2 peptide as liposome vaccine, which could be effectively taken up by DCs and led to increased DCs activation, enhancing the efficacy of tumor vaccine (95).

### 3.2 Enhance lymph node drainage

Lymph nodes (LNs) are small, bean-shaped organs located throughout the body that are an essential part of the immune system. Within the lymph nodes, immune cells such as DCs, macrophages, B cells, and T cells interact and collaborate to initiate and coordinate adaptive immune responses against foreign invaders like viruses, bacteria, and cancer cells (121). DCs residing in peripheral tissues capture antigens and then migrate through afferent lymphatic vessels toward draining lymph nodes under the action of the chemokine receptor CCR7 (122). Antigen-presenting DCs cells that reach the lymph nodes induce the activation of homologous naive T lymphocytes, stimulating and amplifying the immune response of tumor antigen-reactive T lymphocytes (123). After activation in the lymph nodes, the antigen-specific T lymphocytes travel throughout the body via the efferent lymphatic vessels and circulatory system. These T cells can directly or indirectly recognize and respond to the antigen and migrate to the tumor site to eliminate tumor cells containing the same antigen (124).

The immune microenvironment of peritumor lymph nodes, such as tumor-draining lymph nodes (TDLNs), is thought to be immunosuppressive, which can create favorable conditions for cancer cells to migrate to their proliferating lymph nodes and even spread to other organs (125). To achieve a full anti-tumor effect, effective delivery of the vaccine to lymph nodes is required (124). Nanoparticle sizes between 10-50 nm are more likely to be primed to the lymph nodes. A polymeric micelle containing a platinum-based anticancer drug with a diameter of 30 nm was reported to be more effective in preventing tumor lymph node metastasis by better tumor penetration and accumulation compared to nanomicelles with a diameter of 70 nm under similar conditions of stability, zeta potential, drug release rate, and plasma clearance (126). Neutral or negatively charged NPs are more likely to flow freely into lymphatic vessels than positively charged NPs. Researchers constructed phenylboronic ester modified trp2 tumor antigen-containing peptide nanovaccine and demonstrated that TRP2 nanovaccine could be effectively trapped into lymph nodes, taken up by DCs, and induced DCs maturation as one of the results of increasing negative charge (18). Different shapes of nanomaterials lead to different targeting and accumulation effects. Mueller and his colleagues reported that cylindrical (80 × 180 nm) hydrogel platforms, which are more amenable to LNS targeting and aggregation than conventional spherical NPs, can elicit stronger immune responses (127). Improving the ability of peripheral DCs to homing to lymph nodes by surface modification of NPs is also a target for cancer immunotherapy. In addition, since the targeted migration of DCs to lymph nodes requires CCR7 for initiation, achieving high expression of CCR7 via nanovectors is also one of the strategies to enhance lymph node targeting. Encapsulation of OVA and plasmid DNA encoding CCR7 in nanomicelles achieves active DCs targeting and cytoplasmic release of antigen, which not only promotes DCs activation and maturation but also facilitates DCs migration to lymph nodes by increasing CCR7 expression, thereby enhancing CD8^+^ T cell immune responses (109).

Notably, the route of administration of nanovaccine can also affect their organ targeting. Several studies have shown that transdermal administration via microneedles promotes vaccine accumulation in TDLNs, enhances cellular uptake, activates DCs maturation, and enhances Th1 immune response (128–130).

### 3.3 Promote DCs maturation and antigen presentation

DCs are specialized APCs that are crucial in initiating and modulating immune responses (131). They play a key role in the recognition, capture, and presentation of antigens to T cells, which in turn activate effector cells that can eliminate pathogens, infected cells, or cancer cells (132) (**Box 1**). A widely accepted view is that immature DCs promote immune tolerance, while mature DCs stimulate immune responses to foreign antigens (133). More and more nanovaccines are developed with the inclusion of immunostimulatory components to promote DCs maturation, in addition to effective antigen loading. PRRs play a crucial role in DCs maturation and are essential components of innate immunity required to activate DCs, serving as a bridge between innate and adaptive immunity (134). Toll-like receptors(TLRs) are a widely known family of PRRs, and their agonists have been extensively studied as adjuvant components of nanovaccines (135). Zhang et al. used graphene oxide (GO) nanosheets as a carrier and used electrostatic interactions to couple OVA and adjuvant CpG ODN, aTLR 9 agonists, to GO-PEI nanosheets to effectively induce DCs maturation. It significantly inhibited tumor growth and prolonged survival in a mouse model of cholera (136). In recent years, researchers have gained a better understanding of DCs antigen cross-presentation mechanisms, and endosome-targeted nanovaccines are playing an increasingly important role in promoting antigen cross-presentation and DCs maturation. For instance, a TLR7/8 agonist-conjugated nanovaccine (TNV) was synthesized with an ultrasensitive pH-responsive component, iPDPA polymer. This polymer can detect a slight decrease in endosomal pH upon disassembly into monomers. Upon treatment with histone B, the free Toll-like receptor agonist imidazoquinoline (IMDQ) compound is released, which stimulates TLR7/8 localized on the endosomal membrane. This stimulation leads to DCs maturation and elicits specific cellular immunity (105).

**Box 1: DCs exist in two functional states, “immature” and “mature”, and these states are characterized by differences in morphology, phenotype, and function. Immature DCs are specialized in antigen capture and processing (137), whereas mature DCs are specialized in antigen presentation and activation of T cells (138). DCs residing in peripheral tissues digest antigens into peptide fragments via endocytosis to form MHC molecule-antigen peptide complexes (signal 1) (139), after which they migrate to secondary lymphoid tissues while upregulating many co-stimulatory molecules of the B7 family, such as CD80, CD86, (signal 2) (139) and producing a large number of cytokines (signal 3) (139) that drive clonal proliferation and initial immune responses of T lymphocytes (133). There are two classical pathways for antigen presentation by APCs to T lymphocytes. The first pathway is for internalized exogenous antigens and pathogen-derived antigens. They are degraded in endolysosomes, and peptides are loaded onto MHC-II molecules. These molecules are then exposed to the cytoplasmic membrane to activate CD4^+^ T lymphocytes (140). Another pathway for antigen presentation involves endogenous cytoplasmic antigens, which are processed by the cytoplasmic proteasome. The hydrolyzed peptides are loaded onto MHC-I molecules in the endoplasmic reticulum (ER) and then transported to the cytoplasmic membrane for presentation to CD8^+^ T lymphocytes (140). However, this did not explain the fact that DCs not directly infected by pathogens could also activate the production of CTLsuntil the concept of cross-presentation was introduced, whereby the MHC I molecules of DCs could present peptides not only from their proteins but also from exogenous (extracellular) sources to CD8^+^ T lymphocytes (141). Numerous studies have demonstrated that *in vivo*, DCs are the primary cross-presenting APCs (142), underscoring the significance of DC cross-presentation in the development and enhancement of nanovaccines.**

In the context of vaccine development, exogenous antigens are first taken up by DCs via endocytosis and can be further processed and trimmed by resident cysteine protease histone proteases, such as histone S. Alternatively, these antigens can be directly digested by active proteasomes, and the resulting antigenic peptides are loaded onto MHC-I molecules derived from circulating endosomes, leading to activation of CD8^+^ T cells. To enhance the antigen cross-presentation process, one study utilized nanocarriers to attach ovalbumin to substrates of histone S in phagocytosed endosomes, effectively targeting histone S. The results of this study demonstrated that the use of nanocarriers significantly enhanced the efficiency of resident histone-mediated cross-presentation in DCs (143). In addition to targeting specific extracellular receptors on DCs, endosomal escape is another strategy to facilitate antigen cross-presentation. Large intracytoplasmic delivery of antigens can be induced by endosomal escape, which enhances antigen cross-presentation. For instance, bacterial-derived outer membrane vesicles (OMVs) have been used as an mRNA delivery platform to promote endosomal escape. In one study, RNA-binding protein L7Ae and lysosomal escape protein listeriolysin O were surface-modified onto OMVs to deliver mRNA to DCs. The results showed that the vaccine significantly inhibited tumor progression, promoted tumor regression, and established long-term immunity for a certain time (101). Cell-penetrating peptides, which are composed of 4-40 amino acids, are effective tools for delivering mRNA and have been shown to facilitate endosomal escape (144). Wu et al. used cytoplasmic localized internalizing peptide 6 (CLIP6), a specific CCP, to couple to the OVA for direct translocation across the cell membrane (145). The uptake of this nanoparticle by DCs is significantly increased compared to bare OVA and, more importantly, significantly enhances antigen cross-presentation and induces a stronger CTL-mediated immune response with the help of adjuvants. Similarly, researchers synthesized a DC-targeted pH-sensitive liposome nanovaccine, which consisted of a pH-sensitive polymer that became hydrophobic under weakly acidic conditions in endosomes. This property promoted the fusion of liposomes and endosomal membranes, enhancing antigen release to the cytoplasm and improving cross-presentation efficiency by DCs (106).

### 3.4 Remodel the TME

The suppressive effects of the TME often result in reduced infiltration or dysfunction of immune cells such as DCs, and their defective function is one of the key factors responsible for tumor evasion of immune surveillance (146). In the TME, nanovaccines are an attractive tumor treatment that initiates the cancer immune cycle by inducing the immunogenic death of tumor cells and generating a rich pool of tumor antigens (147, 148). Enhancing the function of DCs in response to the characteristics of the TME is crucial in tumor immunotherapy to transform “cold” tumors and metastasis into “hot” tumors (149). In a recent study, a novel charge-reverting polymeric nanomodulator, SPDMCN, was designed to effectively target the TME. In the blood circulation, SPDMCN maintained a negative charge to ensure high stability and aggregation toward the tumor site. Once SPDMCN reached the acidic TME, the surface charge reversed to a positive charge, releasing immunomodulators and enhancing tumor site penetration. Under NIR laser irradiation, SPDMCN generated single linear state oxygen, which induced immunogenic death and released TAAs. This process effectively promoted DCs maturation and antigen-specific CTL infiltration, ultimately leading to a robust anti-tumor immune response (150). Hypoxia is a major feature of most solid tumors and one of the main reasons for the failure of many tumor immunotherapies in clinical practice. Hypoxia can affect the maturation, migration, and Th-cell polarization of functional anti-tumor DCs (151). To overcome this challenge, a potential solution is the use of manganese porphyrin-based multifunctional (Mn-MOF) nanoplatforms with peroxidase-like activity. These nanoplatforms can catalyze the production of O2 from tumor-overexpressed H_2_O_2_ to alleviate tumor hypoxia. The results show that Mn-MOF significantly increased the number of activated CD8^+^ T lymphocytes and mature DCs in tumor tissues, effectively remodeling the tumor immune microenvironment (152). These studies suggest that reversing the immunosuppressive TME can effectively restore the function of DCs and initiate the body’s anti-tumor immune response, which is a promising strategy for tumor immunotherapy.

### 3.5 Stimulate T lymphocytes

Most immunotherapies aim to restore the immune system or more precisely the ability of immune cells to sense and eliminate cancer (153). As an important component of the immune system, CTLs are the key effectors of anticancer immunity, specifically recognizing and killing neoplastic cells (154). Activation of tumor-specific T lymphocytes is dependent on the antigen-presenting function of DCs (155). Zhang et al. developed a thiolated nanovaccine that allows direct cytoplasmic delivery of neoantigens and the Toll-like receptor 9 agonist CpG-ODN. This approach was able to bypass endocytosis/lysosomal degradation, increase the uptake and local concentration of neoantigen and CpG-ODN, activate APCs, and significantly enhance anti-cancer T cell immunity (107). One of the major barriers to DCs initiation and activation of T lymphocytes needs to be overcome is the suppressive immune checkpoint molecules expressed on tumor-infiltrating T lymphocytes, such as CTLA-4 molecule and PD-1. By blocking their expression, it helps to improve the efficiency of T cell activation by DCs vaccines (156, 157). CTLs need to reach tumor tissue to perform effector functions effectively. Studies have shown that local application of STING can promote vaccine-initiated tumor infiltration of CD8^+^ T lymphocytes, overcoming to some extent the dilemma of poor efficacy of tumor vaccination (158).

## 4 Summary

Immunotherapy is an important and effective treatment for oncology. Despite its promising results, the development of safe and effective oncology vaccines remains challenging. Nanotechnology can be used to encapsulate tumor antigens and adjuvants in various types of nanocarriers, allowing modification of their physicochemical properties to enhance targeting of tumor antigens, prolong stability in circulation, achieve high concentration and sustained release at the site of action, and reduce biological toxicity.

Nanovaccine plays an important role in tumor targeting therapy. In addition to targeting specific immune cells or tumor cells, they can also target the immunosuppressive TME. More and more studies have shown that designing nanovaccine reasonably can reverse immunosuppressive TME, like hypoxia (159), acidosis (160), excessive glutathione (161), and hydrogen peroxide (162), etc. to enhance immune cell infiltration and reactivate the body’s responsiveness to tumors.

Tumor development is a long-term and complex process and single therapies are prone to immune tolerance or off-target effects and benefit a small population. Therefore, combination therapy is considered one of the important ways to improve the effectiveness of tumor vaccines. Nanomaterials, because of their modifiability, can be connected with physical or chemical components with different potencies to achieve combination therapy of photodynamic therapy, cytokine therapy, and immune checkpoint blockers with tumor vaccines, which can effectively reduce the generation of toxic side effects, achieve amplified anti-tumor immune effects and establish long-term anti-tumor immune memory.

In summary, nanovaccines have shown encouraging results in experimental studies of tumor immunotherapy. Cost and clinical translation issues should be considered in the design of nanovaccines to provide treatment to patients. And it shouldn’t just focus on innovation. The successful clinical transformation of nanomaterials requires the joint efforts of nanomaterials science, immunology, oncology, and pharmacy to promote and ultimately benefit more tumor patients.

## Author contributions

PW and LZ wrote the concept and design for paper. MY wrote the paper and collected references. XL wrote the revision.The drawings were made by ZQ, DF and XS. All authors contributed to the article and approved the submitted version.
